# The Fate of Nitrate in Intertidal Permeable Sediments

**DOI:** 10.1371/journal.pone.0104517

**Published:** 2014-08-15

**Authors:** Hannah K. Marchant, Gaute Lavik, Moritz Holtappels, Marcel M. M. Kuypers

**Affiliations:** Max Planck Institute for Marine Microbiology, Bremen, Germany; Auckland University of Technology, New Zealand

## Abstract

Coastal zones act as a sink for riverine and atmospheric nitrogen inputs and thereby buffer the open ocean from the effects of anthropogenic activity. Recently, microbial activity in sandy permeable sediments has been identified as a dominant source of N-loss in coastal zones, namely through denitrification. Some of the highest coastal denitrification rates measured so far occur within the intertidal permeable sediments of the eutrophied Wadden Sea. Still, denitrification alone can often account for only half of the substantial nitrate (NO_3_^−^) consumption. Therefore, to investigate alternative NO_3_^−^ sinks such as dissimilatory nitrate reduction to ammonium (DNRA), intracellular nitrate storage by eukaryotes and isotope equilibration effects we carried out ^15^NO_3_^−^ amendment experiments. By considering all of these sinks in combination, we could quantify the fate of the ^15^NO_3_^−^ added to the sediment. Denitrification was the dominant nitrate sink (50–75%), while DNRA, which recycles N to the environment accounted for 10–20% of NO_3_^−^ consumption. Intriguingly, we also observed that between 20 and 40% of ^15^NO_3_^−^ added to the incubations entered an intracellular pool of NO_3_^−^ and was subsequently respired when nitrate became limiting. Eukaryotes were responsible for a large proportion of intracellular nitrate storage, and it could be shown through inhibition experiments that at least a third of the stored nitrate was subsequently also respired by eukaryotes. The environmental significance of the intracellular nitrate pool was confirmed by *in situ* measurements which revealed that intracellular storage can accumulate nitrate at concentrations six fold higher than the surrounding porewater. This intracellular pool is so far not considered when modeling N-loss from intertidal permeable sediments; however it can act as a reservoir for nitrate during low tide. Consequently, nitrate respiration supported by intracellular nitrate storage can add an additional 20% to previous nitrate reduction estimates in intertidal sediments, further increasing their contribution to N-loss.

## Introduction

Human activity has dramatically increased the amount of fixed nitrogen (N) in the environment, to the extent that anthropogenic sources now contribute 156 Tg yr^−1^, almost as much as biological nitrogen fixation in the ocean (177 Tg), and more than biological N_2_-fixation on land (107 Tg) [1], [2]. Worldwide, coastal seas receive a significant amount of this anthropogenic N through riverine run off (48 Tg N yr^−1^) [3], much of which is respired to N_2_ within the sediment by benthic denitrification and anammox. The high rates of these processes within shelf sediments mean that they can account for up to 70% of all sedimentary N-loss [4]. Therefore coastal seas act as a buffer, protecting the open ocean from the impact of anthropogenically derived N [5].

Permeable, coarse grained sediments cover 58–70% of continental shelves and a large part of the coastal zone [6]. Nonetheless, most studies of N-loss in coastal seas have focused on muddy diffusively controlled sediments and have neglected sandy sediments, despite their role as highly efficient biocatalytic filters, in which organic matter inputs from the water column are quickly remineralized [7]. Remineralization is stimulated in permeable sediments by advective pore water transport, which occurs when bottom water currents pass over sediment topography such as ripples, leading to variations in pressure gradients [8]. As a result the sediment is supplied with nutrient and oxygen rich water to penetration depths up to 6 cm [9]–[11]. In addition to high remineralization rates, the conditions present in permeable sediments result in some of the highest potential denitrification rates in the marine environment [12]–[14] and also stimulate aerobic denitrification [15], [16]. The Wadden Sea, which is one of the world's largest intertidal ecosystems, is an example of an environment largely characterized by permeable sandy sediments, in which microbially mediated processes remove at least a third of anthropogenic N-inputs [12], [17]. These inputs are extensive and can be up to 640–820 kt N year^−1^, the vast majority of which enters the Wadden Sea as riverine discharges from the Rhine, Elbe and Weser. These rivers contain high nitrate loads as they represent catchment areas for a large part of the European agricultural run-off [18].

Gao et al. (2010) [15] first reported the occurrence of exceptionally high denitrification rates in the Wadden Sea using a combination of percolation techniques and isotope labeling studies. Despite high rates of denitrification, NO_x_ consumption exceeded N_2_ production by a factor of 2 (22 mmol N m^−3^_sediment_ h^−1^ compared to 8 mmol N m^−3^_sediment_ h^−1^) [12], [15]. It seems unlikely that NO_x_ assimilation alone could account for this discrepancy since incubations were run in the dark, reducing the potential for algal uptake of nitrate [19]–[21]. Indeed, it has been demonstrated that ^15^NO_3_^−^ assimilation into organic matter is negligible in sediments incubated in the dark. Instead, nitrate turnover is almost exclusively controlled by dissimilatory processes such as denitrification and dissimilatory nitrate reduction to ammonium (DNRA) [22].

Denitrification is the best studied pathway of nitrate reduction in permeable sediments; whereas the alternative pathway of DNRA has rarely been investigated and has never been tested using percolation techniques (i.e. when water spiked with the appropriate tracer, in this case ^15^NH_4_^+^, is perfused into the sediment (see [9], [23] for details)). While DNRA consumes NO_x_, it does not produce N_2_ or N_2_O and as such does not contribute to N-loss but rather leads to N-recycling. Therefore, when DNRA is substantial, it can lead to sustained primary production and nitrification [24]. Many microorganisms can perform DNRA, including heterotrophic and chemoautotrophic prokaryotes [25]. Recently eukaryotes, namely diatoms, have also been shown to carry out DNRA, a metabolic process that is associated to intracellular storage of nitrate at millimolar concentrations [26].

In general, intracellular storage of nitrate appears to be common in permeable intertidal sediments and represents a significant pool of NO_x_ which is not measurable in the porewater [27], [28]. Intracellular nitrate pools in sands have largely been attributed to diatoms [29], [30] while in muddy sediments, foraminifera [31] and large vacuoled sulfur bacteria have also been identified storing nitrate at high intracellular concentrations [32]–[34]. Nitrate storage offers an advantage to microorganisms in intertidal permeable systems, where oxygen and nitrate concentrations fluctuate frequently. However, it can also complicate stable isotope studies, as ^15^N-label additions to sediment incubations have been observed to cause nitrate release to the porewater [35], [36], or rapid equilibrations between stored ^14^NO_3_^−^ pools and added ^15^NO_3_^−^ pools [22].

Diatoms, both from the water column and the microphytobenthic (MPB) layer, can contribute significantly to N-uptake and N-loss in permeable sediments [37]. Benthic primary production is enhanced in permeable quartz sands, as they refract light, leading to greater light penetration than in fine grained diffusive sediments [38]. These MPB are subsequently more likely to be buried within permeable sediments during physical disturbance of the sediment [39], [40]. After burial and the onset of anoxic conditions, diatoms have been shown to switch from aerobic respiration to nitrate respiration as a survival mechanism [26]. So far, the overall contribution of these buried MPB to nitrogen cycling is not well understood.

Here we used a non-destructive percolation method, which enabled determination of nitrate conversion processes such as denitrification, DNRA and intracellular storage at high resolution in whole core incubations (Figure S1). Previously, whole core percolation incubations have involved destructive sampling of an entire core for a single time point (e.g. Gao et al., 2012 [12]), while multiple or continuous sampling required slurry incubations (e.g. Gao et al., 2010 [15]). In this study, the use of whole core incubations directly linked to Membrane Inlet Mass Spectrometry (MIMS) enabled immediate determination of oxygen consumption rates and denitrification rates. Subsequent timing and labeling of further isotope experiments were then fine-tuned to provide high resolution data. Thereby we were able assess the fate of nitrate within the sediment by quantifying nitrate reduction to N_2_ and NH_4_^+^ by both eukaryotes and prokaryotes and also determine the role that intracellular nitrate storage plays within permeable sediments.

## Methods

### Sampling site

The Janssand sand flat (13 km^2^) is located in the back barrier area of Spiekeroog Island in the East Frisian Wadden Sea, Germany. The intertidal flat consists of three regions, the upper flat, the slope between the upper flat and the low water line and the low water line. The upper flat, which is the focus of the this study, consists of well sorted silicate sand in the upper 15 cm, with a permeability of 7.2–9.5 x 10^−12^ m^2^, a porosity of 0.35 and a mean grain size of 176 mm [41], [42]. At high tide the flat is covered by 1.5–2 m of seawater for 6–8 h and is exposed to air for 6–8 h during low tide, dependent on tidal range. The sampling site (53.73515′N, 007.69913′E) is on the northeastern margin of the flat around 80 m upslope from the mean low water line. Due to the high permeability of the sand, water is flushed advectively through the sediment in distinct temporal and spatial scales. During inundation, boundary flows force water into troughs on the rippled surface, filtering organic particles and nutrients. The “skin circulation” of porewater occurs at spatial scales in the order of centimeters and temporal scales of minutes to hours [42].

### Sample collection and sediment cores

Sediment sampling for ^15^N-labelled incubations was conducted on the upper flat in autumn, spring and summer on 1 November 2011, 13 April 2012 and 20 July 2012 respectively. The permission to collect sediment samples for scientific purposes was issued by the Nationalparkverwaltung Niedersächsisches Wattenmeer (Wilhelmshaven, Germany). The sampling campaigns did not involve endangered or protected species. Sediment was collected from the upper 5 cm of the sand flat, placed in a prewashed plastic container and returned to the Max Planck Institute, Bremen. Within 4 hours of collection, homogenized sediment was carefully packed into sediment cores, ensuring no bubbles were present.

Cores were constructed of PVC tubing (height 9 cm, I.D. 10.3 cm) and sealed with rubber stoppers. Inflow and outflow ports were provided by boring 0.5 cm I.D. holes through the centre of the rubber stopper, which were fitted with 2 way valves. The internal face of each rubber stopper was milled with radial grooves to create radial pressure and flow through the cores. To prevent sediment from filling the grooves and obstructing flow, they were covered with a fine mesh filter (500 microns, Hydra-BIOS, Germany). Cores and supply seawater were held at 19°C for the entire experiment.

After sediment sampling and packing into cores, the cores were allowed to equilibrate overnight, during which time, aerated site seawater was pumped through them on a simulated tidal cycle consisting of 8 subsequent cycles of 30 minutes with pumping (at 2.5 ml min^−1^) and 15 minutes without pumping, followed by 6 hours without additional water supply. All incubations were carried out after 24 hours of equilibration. Homogenization of sediment has previously been shown to have little effect on nitrogen cycling rates, as observed in Gao et al., (2010) [15].

### Incubations

Core incubations were carried out with the intention of following the transformations of nitrate within the sediment over time, firstly when the sediment was oxic, and secondly as the sediment became anoxic. This simulates the *in situ* conditions, whereby the porewater is oxic when nitrate is first advected into the sediment, but becomes anoxic over time as the porewater ages (in respect to the time it entered the sediment). To achieve this, the entire porewater volume was exchanged in each core with aerated porewater and then sampled over time (see details below). In each season experiments were carried out on 3 replicate sediment columns. Incubations were started by amending 600 ml of aerated site seawater with labeled and unlabeled substrate dependent on the process being investigated (Table 1). Water was then pumped through the core from the bottom using a peristaltic pump at 30 ml min^−1^. After 20 minutes the entire porewater volume had been replaced (as confirmed by breakthrough curves using NaBr as an inert tracer, see Fig. S2). The inflow port was then closed, a sampling port was connected to the 2-way valve at the bottom of the core and porewater was then sampled using one of two methods (Fig. S1).

**Table 1:** Summary of sampling dates, conditions, and ^15^N incubation experiments conducted.

Sampling Date	Substrate Additions	Sampling	Processes Targeted
	(*µM*)	method^a^	
November 1st 2011	^15^NO_3_^−^ (50)	MIMS	O_2_ consumption, denitrification
	^15^NO_3_^−^ (50)	Exetainers	Denitrification, DNRA
	Unamended	Exetainers	Storage
	^15^NO_3_- (50) +200* µg* cycloheximide*	Exetainers	Denitrification, DNRA
	200 ug cycloheximide	Exetainers	Euk. and Prok. rates
April 13rd 2012	^15^NO_3_^−^ (50)	MIMS	O_2_ consumption, denitrification
	^15^NO_3_^−^ (30)	Exetainers	Denitrification, DNRA
	Unamended	Exetainers	Storage
	^15^NO_3_^−^ (30) +200* µg* cycloheximide*	Exetainers	Denitrification, DNRA
	200 ug cycloheximide	Exetainers	Euk. and Prok. rates
July 22nd 2012	^15^NH_4_^+^ (50) + ^14^NO_2_^−^ (100) + ^14^NO_3_^−^ (100) + ATU (86)	Exetainers	Anammox**
	^15^NO_3_^−^ (50)	MIMS	O_2_ consumption, denitrification
	^15^NO_3_^−^ (50)	Exetainers	Denitrification, DNRA
	Unamended	Exetainers	Storage
	^15^NO_3_^−^ (50) +200* µg* cycloheximide*	Exetainers	Denitrification, DNRA
	200* µg* cycloheximide	Exetainers	Euk. and Prok. rates

a For further detail see description in text.

* Before incubation sediment cores were additionally preincubated for 6 hours with porewater containing 200* µg* L^−1^ cycloheximide.

** The sediment cores used in this incubation were not used for further incubations.

In the first instance the sampling port was connected via a piece of tygon tubing to a membrane inlet mass spectrometer (MIMS; GAM200, IPI). Porewater was pumped across the membrane by a peristaltic pump placed downstream of the membrane and at a speed of 500* µl* min^−1^ - equivalent to a porewater flow velocity of around 1 cm h^−1^. This allowed simultaneous online measurements of mass 28 (^14^N^14^N), 29 (^14^N^15^N), 30 (^15^N^15^N), 32 (O_2_), 40 (Ar). Oxygen consumption rates, ^29^N_2_ and ^30^N_2_ production rates showed low standard deviation between sediment cores, indicating that this method gives replicable results.

In the second instance, porewater was collected by opening the 2 way valve on the bottom of the core and letting porewater flow directly into 6 ml exetainers (Labco Ltd, High Wycombe, UK), prefilled with 100* µl* saturated HgCl_2_. 1.5 ml of porewater was discarded initially at each time point to flush the tubing between the core and the sediment and 12 time points were sampled over 180 minutes (the total volume sampled was therefore 90 ml per incubation, less than one third of the entire porewater volume in the core and representing a flow velocity around 2–3 cm h^−1^). In both cases sampled porewater was replaced passively with unamended sea water from the top of the core. In order to examine storage driven rates of N-removal, after the last time point the entire porewater volume within the sediment core was exchanged for unlabeled water and over 4 hours the accumulation of the same labeled compounds was followed (i.e. ^15^N-N_2_ and ^15^NH_4_^+^). Following this the cores were preincubated with cycloheximide an inhibitor of eukaryotic protein synthesis (an inhibitor of eukaryotic protein synthesis [43]) for six hours and both incubations were repeated. The entire sampling scheme is shown in Figure S1.

In July 2012, further incubations were carried out on 3 parallel sediment cores to determine ammonia oxidation rates. The incubation procedure was the same, except that the aerated seawater was amended with 100 µmol L^−1^ of ^15^NH_4_^+^ and 150 µmol L^−1^ of ^14^NO_2_^−^.

### Oxygen measurements

In April and July the oxygen concentration of the porewater at each time point was determined in the exetainer immediately after filling using an O_2_ microsensor. Oxygen microsensors were constructed as described in Revsbech (1989) [44]. A two point calibration was performed prior to sampling using air saturated seawater and N_2_ degassed seawater. There were only small differences between oxygen consumption rates when determined from MIMS or by direct microsensor measurement in exetainers. Therefore, for the samples from autumn where no microsensor measurements were available, O_2_ concentrations were determined from the initial MIMS incubation.

### Intracellular nitrate storage determination

Sediment and porewater was collected on October 24 2013 from the upper sand flat and approximately 5 m downslope for the determination of both porewater and intracellularly stored nitrate. These parts of the sand flat had been exposed to the air for approximately 120 minutes and 90 minutes respectively. Rhizon samplers [45] were used to directly sample porewater from the top 3 cm of sediment (5 samples per location). Approximately 5 mL of sediment was collected concurrently and added to a 50 mL falcon tube along with 3 mL NaCl solution (adjusted to the *in situ* salinity of 33) and 400* µl* saturated HgCl_2_ (5 samples per location). All samples were kept on ice and upon return stored at −20°C upon return until further analysis. Intracellular nitrate concentrations within the sediment were determined using the method of Stief et al. (2013) [46]. Briefly sediment samples were shock-frozen in liquid nitrogen for 5 min, and then heated to 90°C in a water bath for 10 min, a process which was repeated 3 times. NO_x_ was determined in all samples as detailed below. Diatom abundances within the upper layer of the sediment were estimated using the method described in Ehrenhauss et al. [40], briefly, to separate diatoms from the sediment, 1 ml of sediment was suspended 5 times in 5 ml of artificial seawater and the supernatant collected after 15 s of deposition before being filtered on to 0.2 µm black membrane filters. Counts were carried out immediately on 5 parallel filters in 30 randomly picked counting grids using a Zeiss™ Axioplan 2 epifluorescence microscope. Diatoms counts were split into 4 size classes based on morphology and the average biovolume of each size class was determined using the calculations of Hillebrand et al. [47]. Foraminifera abundance was estimated using the method described in Goineau et al. [48].

### Isotopic and nutrient analyses

2 ml of porewater within each exetainer was replaced with a helium headspace and allowed to equilibrate for at least 24 hours. Subsequently the isotopic N composition of N_2_ and N_2_O gas in all exetainers was determined by GC-IRMS (VG Optima, Manchester, UK). Afterwards, ^15^NO_2_^−^ and ^15^NO_3_^−^ concentrations were determined in subsamples of all samples, after conversion to N_2_ by sulfamic acid addition or cadmium reduction/sulfamic acid addition (after NO_2_ removal) respectively [49]. ^15^NH_4_^+^ was determined in subsamples after oxidation with hypobromite to N_2_ [50], [51]

Concentrations of ^45^N_2_O, ^46^N_2_O, ^29^N_2_ and ^30^N_2_ were calculated from the excess relative to air, explained in detail in Holtappels et al. [52] and rates were calculated from the slope of linear regression of ^15^N-concentration as a function of time. Only significant and linear production or consumption of ^15^N-species was considered (*t*-tests, *p*<0.05; R^2^>0.75).

Combined nitrate and nitrite (NO_x_) concentration within each exetainer was determined by a CLD 60 Chemiluminescence NO/NO_x_ analyzer (Ecophysics) after reduction to NO with acidic vanadium (II) chloride [53]. Total NH_4_^+^ was determined either by flow injection analysis [54] or photometrically [55], dependent on whether samples contained HgCl_2_ or were filtered, respectively.

### Rate determinations

Linear denitrification rates were determined over time within the sediment cores both while oxygen was still present and after it had been consumed. Rates were determined from the production of ^29^N_2_ (p^29^N_2_) and ^30^N_2_ (p^30^N_2_) according to Thamdrup and Dalsgaard [56]:
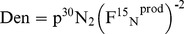
(eq 1)


Where F^15^_N_^prod^ is the labeling percentage of nitrate which was calculated from the production of ^29^N_2_ and ^30^N_2_: 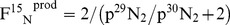
(eq 2)


The labeling percentage of nitrate can also be calculated from the initial concentrations of labeled and unlabeled nitrate (F^15^_N_^added^)



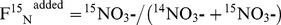
(eq 3)


F^15^_N_^prod^ and F^15^_N_^added^ can deviate during the incubation if processes other than denitrification either add to the ^29^N_2_ (anammox) or change the ^14^NO_3_^−^ pool and thus the fraction of labeled nitrate. Therefore we compared F^15^_N_^prod^ and F^15^_N_^added^.

DNRA rates were determined from the labeling percentage of nitrate (eq 2) and the production of ^15^NH_4_^+^ (p^15^ NH_4_^+^): 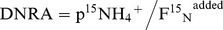
(eq 4)


Eukaryotic and prokaryotic rates for all processes were determined by subtracting the rates obtained in the incubation amended with ^15^NO_3_^−^ and cycloheximide (Inc_cyc_) from the rates obtained in the incubation amended with only ^15^NO_3_^−^ (Inc_1_)
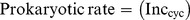
(eq 5)




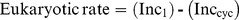
(eq 6)


Mass balances were performed on incubations to which ^15^NO_3_^−^ was added and exetainer sampling performed. At the end of the incubation, when ^15^N-N_2_ production had ceased and ^15^NO_3_^−^ was no longer detected, the concentration of ^15^N present as ^15^NH_4_^+^, ^15^N_2_O, and ^15^N-N_2_ was measured and compared to the ^15^NO_3_^−^ concentration of the added seawater (measured before it was pumped into the sediment. Additionally, upon completion of the incubation to which no ^15^NO_3_^−^ was added, the pools of ^15^NO_3_^−^, ^15^NO_2_^−^, ^15^NH_4_^+^, ^15^N_2_O, and ^15^N-N_2_ were summed up and included in the mass balance.

In July 2012, ammonia oxidation rates were determined from the production of ^15^NO_2_^−^ after the addition of ^15^NH_4_^+^ and ^14^NO_2_^−^. A linear production of ^15^NO_2_^−^ occurred during the first 40 minutes of the incubation. Rates were corrected by the labeling percentage of ammonium at the start of the incubation (F^15^_NH4+_^added^) and the production of ^15^NO_2_^−^ (p^15^NO_2_^−^) (eq 7)
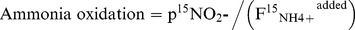
(eq 7)


## Results

### Oxygen consumption rates

**Figure 1:**
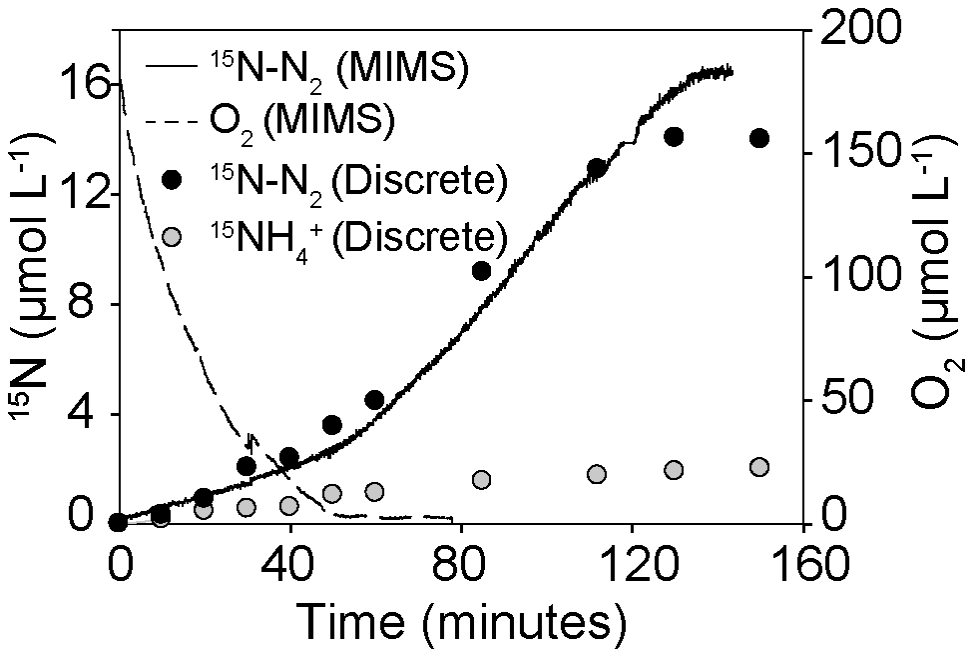
Comparison of incubation methods. Incubations were carried out using either membrane inlet mass spectrometry (MIMS) which allowed for continuous online measurements or by discrete porewater sampling into exetainers and subsequent measurement on a GC-IRMS. The example shown is taken from a replicate during the spring incubations directly after the addition of ^15^NO_3_^−^.

Online Membrane Inlet Mass Spectrometry (MIMS) was used to determine O_2_ consumption rates (OCR) in parallel to ^15^N-N_2_ production rates (Fig. 1). Volumetric OCR were highest in autumn (459±60* µm*ol L^−1^ h^−1^) and lower during spring (271±51* µm*ol L^−1^ h^−1^) and summer (292±23* µm*ol L^−1^ h^−1^). The online MIMS was subsequently used as a reference for later incubations without MIMS to ensure that samples were collected until oxygen was entirely consumed and ^15^N-N_2_ production had ceased.

### Denitrification rates

**Figure 2:**
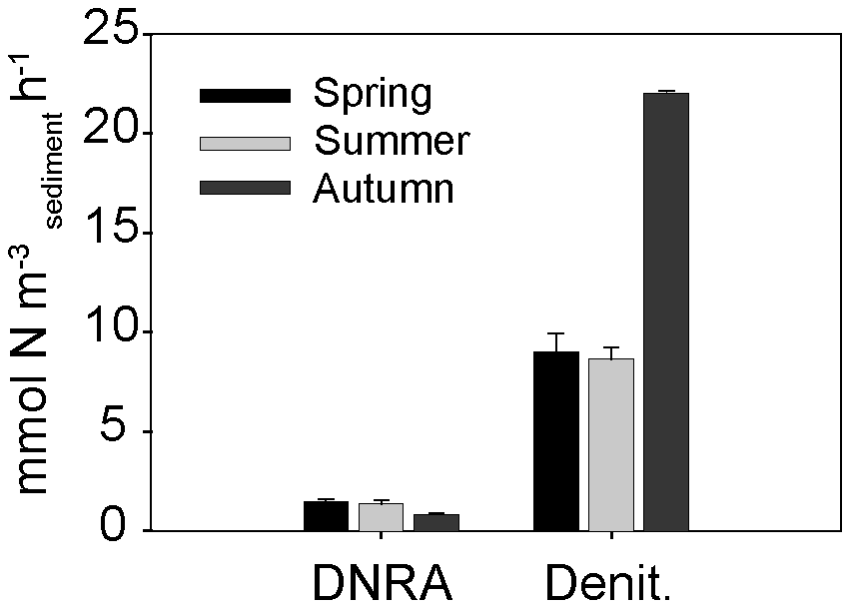
Rates of nitrogen cycling processes determined over 3 seasons. Anaerobic dissimilatory nitrate reduction to ammonium (DNRA) and denitrification (Denit.) rates from the incubation amended with ^15^NO_3_^−^. Rates were determined from linear production slopes after oxygen had been consumed. Error bars are SD (n = 3)

Subsequent to the MIMS incubation, production of ^15^N-N_2_ and ^15^NH_4_^+^ were determined from ^15^NO_3_^−^ amendment experiments in which porewater from the bottom of the core was subsampled discretely into exetainers and measured by GC-IRMS. ^15^N-N_2_ and ^15^NH_4_^+^ concentrations in the subsampled porewater revealed that denitrification occurred under oxic and anoxic conditions (Fig. 1). They also showed that denitrification was the dominant nitrate sink within the sediment in all seasons (Fig. 2). In autumn anaerobic denitrification rates were 22.8±2.9 mmol N m^−3^_sediment_ h^−1^, more than double those in spring (8.65±0.6 mmol N m^−3^_sediment_ h^−1^) and summer (9.02±0.9 mmol N m^−3^_sediment_ h^−1^) (Fig. 2). The anaerobic denitrification rates of individual sediment cores were positively correlated with oxygen consumption rates in all seasons. Furthermore, denitrification occurred in the presence of oxygen in all incubations (Fig. 1), however oxic denitrification rates were 75–95% lower than the anaerobic denitrification rates, i.e. those measured upon the sediment becoming anoxic (Table S1). A further set of incubations carried out on the same sediment were amended with ^15^NO_3_^−^ and cycloheximide, a eukaryote inhibitor. In the cycloheximide incubations, the only change that could be observed in denitrification rates was during autumn, when a slight decrease in rates was observed (data not shown).

### DNRA rates

**Figure 3:**
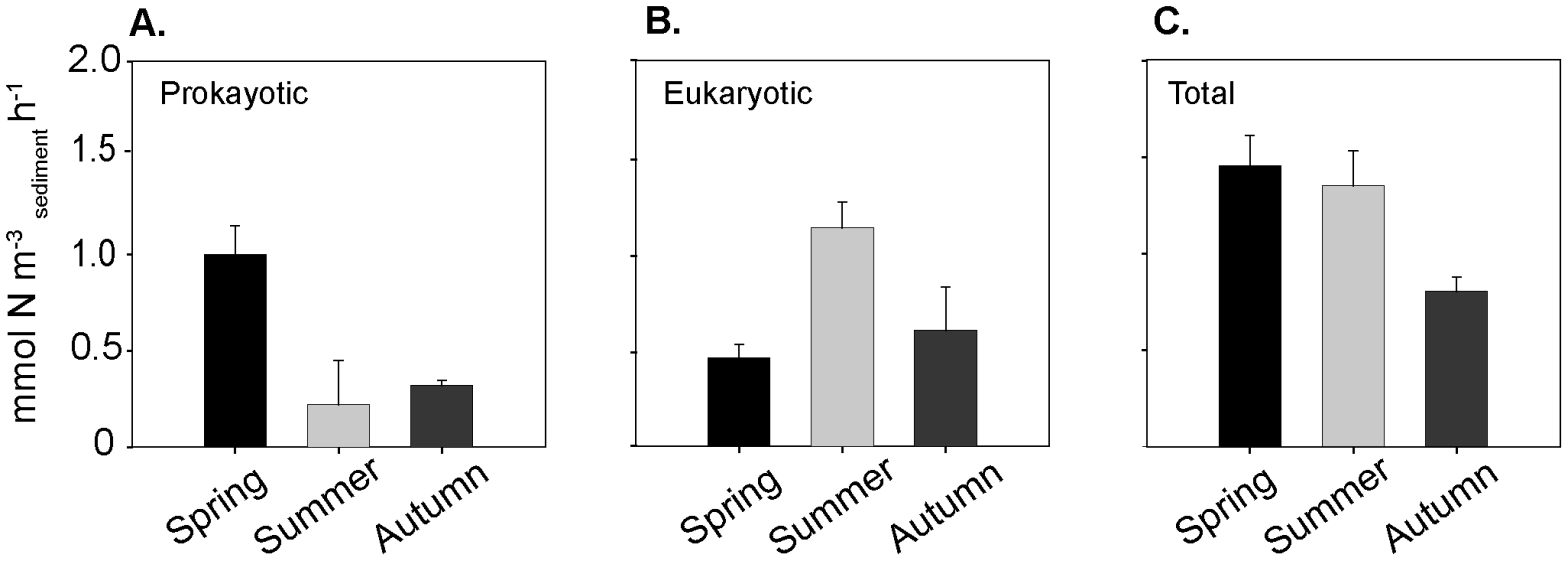
Seasonal patterns in DNRA rates mediated by the prokaryotic and eukaryotic community. A) Eukaryotic DNRA rates B) Prokaryotic DNRA rates C) Overall DNRA rates. Total rates were determined from the incubation amended with ^15^NO_3_^−^. Prokaryotic rates were determined after addition of the eukaryote inhibitor cycloheximide. Eukaryotic rates were calculated by subtracting prokaryotic rates from total rates. Error bars are SD (n = 3)

DNRA rates were determined from the production of ^15^NH_4_^+^ in the ^15^NO_3_^−^ amendment experiments and occurred in all seasons (Fig. 2). DNRA rates were detectable in the presence and absence of oxygen, with oxic rates 60–90% lower than anoxic rates (Table S1). In the ^15^NO_3_^−^ amendment experiment, the only significant seasonal difference in DNRA rates was observed during autumn, when rates were lower. However, the subsequent ^15^NO_3_^−^ + cycloheximide amendment allowed us to determine the contribution of eukaryotes and prokaryotes to DNRA separately. Prokaryotic DNRA rates were higher in summer, while eukaryotic rates were higher in spring (Fig. 3).

### Anammox rates

Anammox could not be detected from incubations in summer which were amended with ^15^NH_4_^+^, ^14^NO_2_^−^, ^14^NO_3_^−^ and allylthiourea. Although incubations for anammox were not carried out in spring or autumn, the ^15^NO_3_^−^ amendment experiment indicated that N_2_ due to anammox was insignificant in these seasons as well. If anammox (NH_4_^+^ + NO_2_^−^ → N_2_), was occurring to any significant extent then ^29^N_2_ production (^14^NH_4_^+^ + ^15^NO_2_^−^ → ^29^N_2_) within the incubation would be higher than expected if only denitrification were reducing ^14+15^NO_3_^−^. This would cause the ratio of ^29^N_2_ and ^30^N_2_ produced (F^15^_N_^prod^) to be lower than expected from the NO_3_^−^ labeling fraction (F^15^_N_^added^). When F^15^_N_^added^ was compared to F^15^_N_^prod^ they did not vary by more than 1.5% at each time point during the anoxic phase of incubations in spring and autumn. Taken together these results indicate that anammox did not occur to any significant extent in any season.

### N-Isotope equilibration in summer

**Figure 4:**
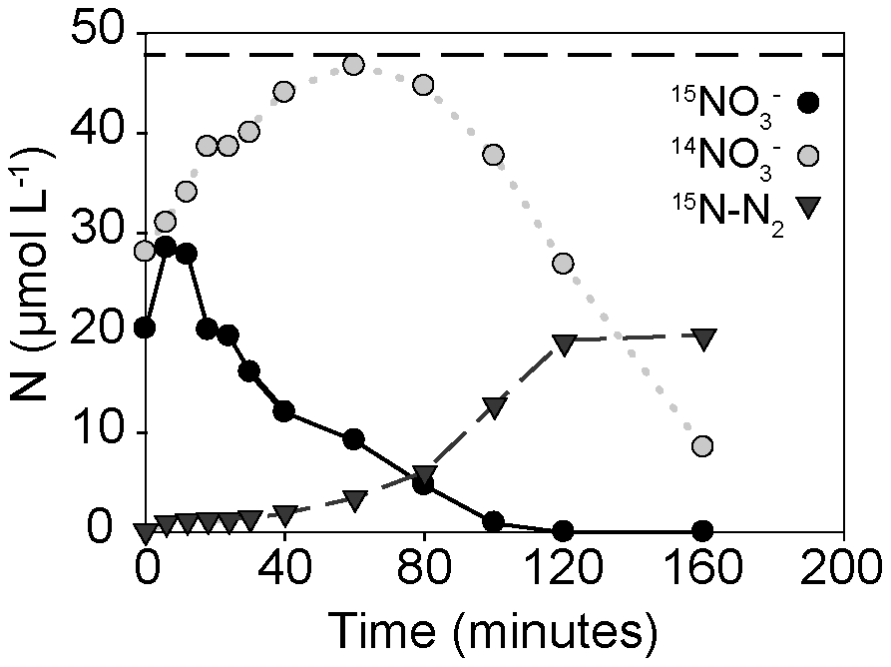
Exchange of ^15^NO_3_^−^ and ^14^NO_3_^−^ over time in summer incubations. The dashed line indicates the initial amount of ^15^NO_3_^−^ added to the incubation

In summer, when no anammox was detected, F^15^_N_^prod^ was significantly lower than F^15^_N_^added^ throughout the ^15^NO_3_^−^ amendment experiment. The difference in F^15^_N_^prod^ and F^15^_N_^added^ was a result of a large initial decrease in the ^15^N labeling percentage of NO_3_^−^ in the porewater (Fig. 4). Almost 35% of the added ^15^NO_3_^−^ was no longer present in the porewater when sampling began, whereas ^14^NO_3_^−^ concentrations were higher in the porewater than the ^14^NO_3_^−^ concentrations measured in the seawater before it was percolated through the sediment. ^14^NO_3_^−^ concentrations continued to increase during the first 30–40 minutes of the incubation, this occurred at a rate of 21.2 µmol L^−1^ (±4.2 SD). Ammonia oxidation rates determined in parallel core incubations were 0.20 µmol L^−1^ (±0.11 SD), too low to account for the increase. Therefore ^14^NO_3_^−^ appeared to equilibrate with ^15^NO_3_^−^. The decrease in labeling percentage occurred in all three summer replicates and represented an apparent ‘loss’ of around 40% of the added ^15^NO_3_^−^ which appears to have been exchanged with ^14^NO_3_^−^ in the initial 40 minutes of the incubation.

### Intracellular nitrate storage

**Figure 5:**
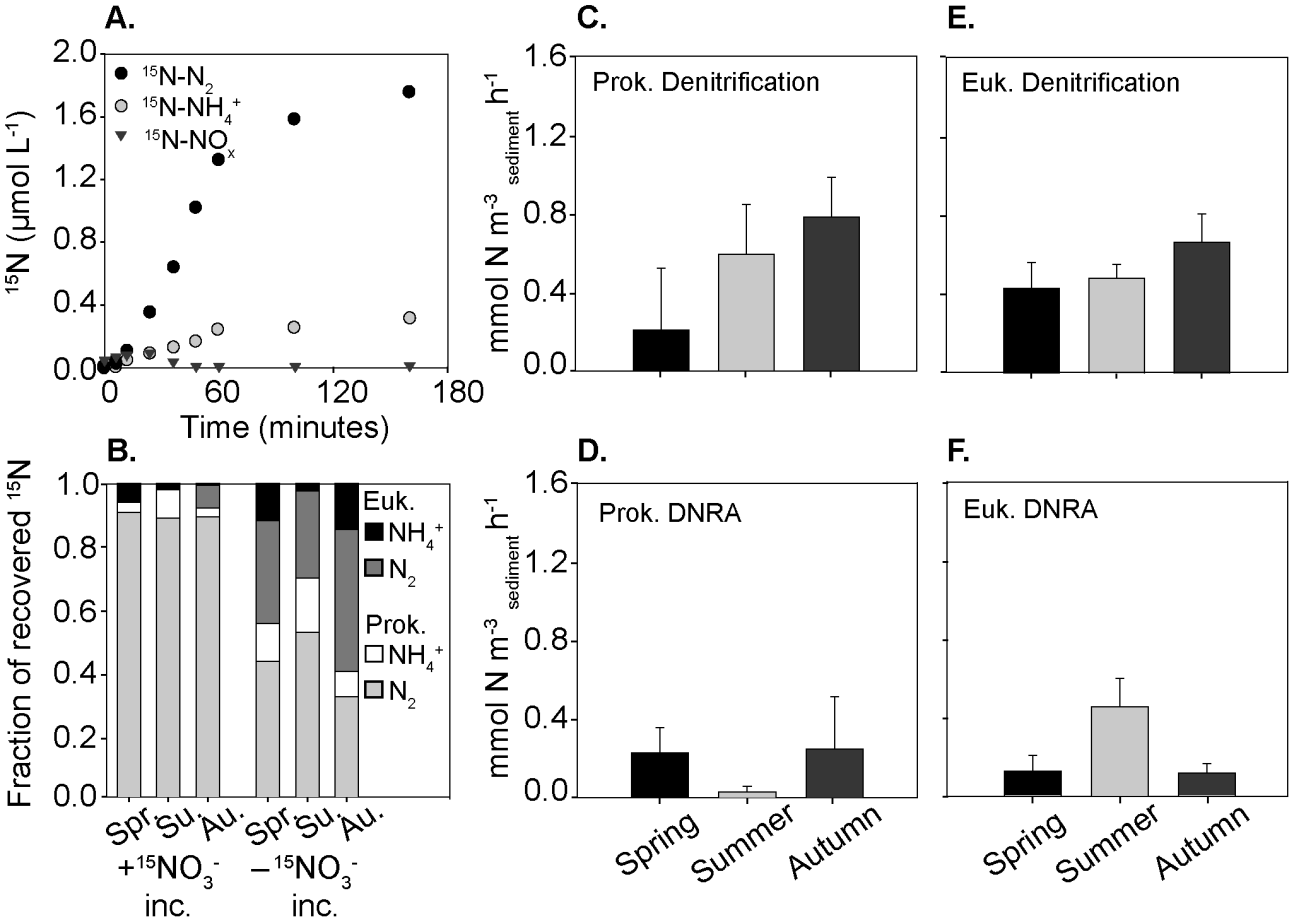
Respiration of stored intracellular nitrate by eukaryotes and prokaryotes. A) Example of the appearance of ^15^N labeled compounds stored in the sediment during the secondary incubation to which no label was added (example from spring incubations) B) Comparison of the overall fraction of ^15^N recovered as either N_2_ or NH_4_^+^ in both the ^15^NO_3_^−^ incubation and the subsequent incubation with no additional ^15^NO_3_^−^ and the partitioning between eukaryotes and prokaryotes C) Eukaryotic denitrification rates E) prokaryotic denitrification rates D) eukaryotic DNRA rates F) prokaryotic DNRA rates. Error bars are SD (n = 3).

In all seasons (including summer, when the 40% ^15^NO_3_^−^ “loss” due to isotope equilibration effects were taken into account), ^15^NO_3_^−^ consumption still exceeded ^15^NH_4_^+^ and ^15^N-N_2_ production in the ^15^NO_3_^−^ amendment experiment. To see whether there had been ^15^NO_3_^−^ uptake into the intracellular pool which was not identified by the isotope equilibration analysis, we performed another incubation after all the ^15^NO_3_^−^ had been consumed and ^15^N-N_2_ production had ceased. In this further incubation we exchanged the entire porewater volume within the sediment core with oxic seawater to which no ^15^NO_3_^−^ had been added. No ^15^NO_3_^−^ or ^15^NO_2_^−^ could be detected in the porewater of the sediment core within the first 5 minutes of this incubation. Subsequently, oxygen consumption (Figure S3) and the production of ^15^NO_x_, ^15^N-N_2_ and ^15^NH_4_^+^ were observed (Fig. 5a). ^15^NO_x_ accumulated after to low concentrations until 10 minutes, but was then consumed rapidly. This accumulation of ^15^NO_x_ was too small to account for the overall production of ^15^N-N_2_ and ^15^NH_4_^+^ observed during the same time period. Therefore another pool of ^15^NO_x_ must have been present within the sediment, which we attributed to intracellularly stored ^15^NO_3_^−^. The intracellularly stored nitrate supported both denitrification and DNRA, however the rates of these two processes and the fraction carried out by eukaryotes and prokaryotes differed when compared to the incubation where ^15^NO_3_^−^ was present in the porewater (Fig. 5b).

The total denitrification rates supported by intracellularly stored ^15^NO_3_^−^ were always higher than the co-occurring DNRA rates, although this was only significant in summer and winter (student t-test, df = 4 p = <0.05 in summer and <0.01 in winter). Overall the storage driven rates of denitrification and DNRA were ∼15% and 30% respectively when compared to rates determined previously in the ^15^NO_3_^−^ amendment experiment. Around 50% (48.5±18.3 s.d.) of the storage driven denitrification rates could be attributed to eukaryotes (Fig. 5c and 5e). Prokaryotes were mainly responsible for storage driven DNRA in summer, whilst in spring and autumn the contributions of prokaryotes and eukaryotes to DNRA were similar (Fig. 5d and 5f).

### *In situ* concentrations of intracellular nitrate storage

To investigate whether intracellular storage of nitrate is relevant in the environment, we determined the concentration of nitrate stored intracellularly *in situ* in early autumn 2013. Porewater NO_x_ concentrations were on average 4.4* µm*ol L^−1^ (±1.0 s.d.) 90 minutes after sediment exposure by low tide and 2.5* µm*ol L^−1^ (±1.2 s.d.) 120 minutes after exposure. Concentrations of nitrate stored intracellularly were 29.5* µm*ol L^−1^ (±3.5 s.d.) in the sediment exposed by low tide for 90 minutes, and 27.5* µm*ol L^−1^ (±6.6 s.d.) in sediment exposed for 120 minutes. On average therefore, concentrations of nitrate stored intracellularly were between 6 and 10 times higher than average porewater nitrate concentrations.

To examine whether the eukaryotic community within the sediment could have accounted for the observed intracellular nitrate storage, we determined the abundance of diatoms and foraminifera. We found foraminifera at abundances of around 8 (±4 s.d.) cm^−3^_sediment_, these were identified as belonging to the genus *Ammonia*. Diatoms were found in abundances of 2.7 (±0.3) x 10^4^ cm^−3^_sediment_. Four classes of diatoms were identified within the sediment which we described as 1) large pennate, 2) chain forming short pennate, 3) chain forming long pennate and 4) other small. These 4 classes of diatoms comprised 55% (±14), 14% (±9), 25% (±15) and 7% (±7) of the total diatom count, respectively. The average biovolumes of the same 4 classes were 3700 µm^3^ (±1240), 930 µm^3^ (±420), 1200 µm^3^ (±250) and 519 µm^3^ (±310), respectively. Cell-volume-specific intracellular nitrate concentrations calculated using the total intracellular nitrate concentrations measured and the average cell volume were therefore 60 mmol L^−1^ diatom. This is well within the range of nitrate that diatoms are known to be capable of storing, therefore diatoms could have stored enough nitrate to account for the intracellular concentrations measured.

## Discussion

We used a non-destructive percolation method to follow transformations of nitrate within permeable sediments from the intertidal Wadden Sea. By amending seawater with ^15^NO_3_^−^ and exchanging it with porewater in a sediment core we could quantify denitrification rates at high temporal resolutions (12 time points over 2–3 hours; Fig. 1). With this method, we observed volumetric denitrification rates ranging from 8–23 mmol N m^−3^_sediment_ h^−1^. These rates represent a two to five fold increase in denitrification relative to rates previously reported from the same site [12]. Gao et al. (2012) used whole core incubations percolated to 5 cm depth with 50 µmol ^15^NO_3_^−^ L^−1^ that were subsequently sacrificed at a given time point, and therefore were restricted to at most 5 time points per incubation. In their incubations, nitrate appeared to be consumed before 4 time points were sampled, which could have led to underestimated denitrification rates. We added the same amount of ^15^NO_3_^−^ (30–50* µm*ol L^−1^) as Gao et al., (2012) but the higher sampling frequency ensured that rates were determined from when linear production was taking place. The consistently higher denitrification rates in this study indicate that the high N-loss estimates for the Wadden Sea reported in Gao et al., 2012, (750 mmol N m^−2^ yr^−1^) might be conservative.

### Denitrification as a nitrate sink

N-loss in the form of N_2_ was the main sink of nitrate in intertidal permeable Wadden Sea sediments. Denitrification was revealed to be the main process by which N-loss occurred, as the combination of ^15^NO_3_- incubations, production ratios of ^29^N_2_ and ^30^N_2_, as well as ^15^NH_4_^+^ incubations showed that anammox was insignificant. These results agree well with previous results from this study site and the Wadden Sea, where ^15^N-labelling experiments have indicated anammox rates are <1% of denitrification [12], [57] and metagenomic analysis has revealed that anammox bacteria are of low abundance (pers. comm. M. Strous).

While denitrification was always the dominant sink of nitrate in the sediment, rates of N-loss differed seasonally. Denitrification rates were double in autumn in comparison to spring or summer (Fig. 2). The seasonal changes in denitrification rates could have resulted from seasonal differences in organic matter limitation, nitrate concentrations or the composition of the microbial community [58]. From our results, we suggest that nitrate and organic matter may both have played a role within our sediments; there was a weak positive correlation between denitrification rates and average seasonal water column nitrate concentrations (r^2^ = 0.67 *p* = <0.01) and a strong positive correlation between denitrification rates and measured oxygen consumption rates (r^2^ = 0.76 *p* = <0.01).

Oxygen consumption rate (OCR) is a proxy for labile organic matter (OM) decomposition [59]. Thus, the decreased volumetric OCR in spring and summer indicate that those seasons had less available labile OM compared to autumn. As OM is a substrate for denitrification, it can limit denitrification rates [60]. Therefore lower summer OM availability could have been responsible for the relatively lower spring and summer denitrification rates. Initially this seems surprising, as OM availability in the water column is higher in spring and summer due to increased pelagic primary production. However, previous studies have shown that despite high water column concentrations of dissolved organic carbon and particulate organic carbon (DOC and POC), summer concentrations of DOC in the surface layer of permeable sediments are comparatively low [61], [62]. This is a result of reduced wind and wave action during summer, which leads to decreased pore water filtration and limits the transport of POC into the sediment. In contrast, during autumn, labile DOC distributions in the surface layer of permeable sediments are at their highest [42], [61]. This is due to high winds, which increase advective porewater flow, thereby enhancing the transport of POC into the surface layer of the sediment, whereupon they are retained by sediment filtration and provide a source of new DOC.

### DNRA as a nitrate sink

In the ^15^NO_3_^−^ amendment experiment, ^15^NO_3_^−^ was reduced to ^15^NH_4_^+^ as well as ^15^N-N_2_, indicating that dissimilatory nitrate reduction to ammonium (DNRA) was occurring (Fig. 1). DNRA was a smaller sink for nitrate than denitrification, with DNRA rates accounting for around 15% of total nitrate reduction rates (DNRA + Denitrification) (Fig. 2). The DNRA rates are comparable to those measured in a diverse range of muddy shelf sediments, including the Baltic sea [63], East China Sea [36], temperate and tropical estuaries [64]–[66] and fjords [67], [68]. However, as denitrification rates are comparatively much higher in the Wadden Sea, the contribution of DNRA to total nitrate reduction measured during this study is lower than in other continental shelf sediments.

The rate of nitrate reduction to ammonium varied seasonally within the sediment, whereby total DNRA rates were higher in spring and summer and significantly lower in autumn. However, different seasonal patterns emerged when prokaryotic and eukaryotic DNRA rates were considered seperately (Fig 3). Prokaryotic DNRA rates were highest in summer (Fig 3b) while eukaryotic rates were highest in spring (Fig. 3a). The high prokaryotic rates of DNRA in summer agree well with the observations of a number of previous studies and may have resulted from a combination of factors; 1) DNRA increases when the ratio of electron donor to acceptor increases [68], [69], as is the case in the Wadden Sea during summer when NO_3_^−^ concentrations in the water column are low in comparison to OM. 2) Based on growth yields, increased summer temperatures and low NO_3_^−^ concentrations have been shown to allow NO_3_^−^ ammonifiers to scavenge NO_3_^−^ more efficiently, and gain more energy per mole of nitrate than denitrifiers [70]. 3) DNRA may also have been more favourable during summer due to enhanced sulfide concentrations in surface sediments at the study site [71]. High sulfide concentrations can inhibit denitrification, and as such, the presence of high sulfide concentrations have been reported to favour DNRA over heterotrophic denitrification [64], [72], [73].

The controls upon eukaryotic DNRA are less well defined than those in prokaryotes. We can speculate however, that high eukaryotic DNRA rates in spring resulted from the settling and burial of diatoms after the spring bloom [10], [40], [74]. Upon burial diatoms have been shown to switch to anaerobic respiration as a survival mechanism at the onset of dark and anoxic conditions [26]. Eukaryotic nitrate respiration is increasingly reported as an important pathway in the nitrogen cycle (See [75] for review), and is usually associated with intracellular storage of NO_3_^−^ or NH_4_^+^ [29], [30], [76]. Therefore, to investigate the potential importance of eukaryote-associated nitrate respiration within Wadden Sea permeable sediments, we looked for evidence of intracellular nitrate storage *in situ*.

### Intracellular storage of nitrate *in situ*

To determine how much nitrate is stored *in situ* within permeable Wadden Sea sediments, we compared porewater nitrate concentrations with intracellular nitrate concentrations. This revealed that shortly after low tide, intracellular nitrate concentrations within the sediment were six fold higher than the porewater concentrations. Benthic diatoms and foraminifera were present at the study site and could have been responsible for this storage. Both diatoms and foraminifera are capable of storing nitrate intracellularly to concentrations orders of magnitude higher than the surrounding porewater, in fact laboratory studies have demonstrated that cell volume specific accumulation can be as high as 450 mmol L^−1^ in diatoms [77] and 570 mmol L^−1^ in foraminifera [31]. If we were to assume that only the diatoms that we identified within the sediment were responsible for intracellular nitrate storage, then, based on diatom cell counts and biovolume estimates, this would make cell specific intracellular nitrate storage in the range of 60 mmol L^−1^, a concentration well within the range identified in laboratory studies.

### Intracellular storage as a short-term sink of nitrate

**Figure 6:**
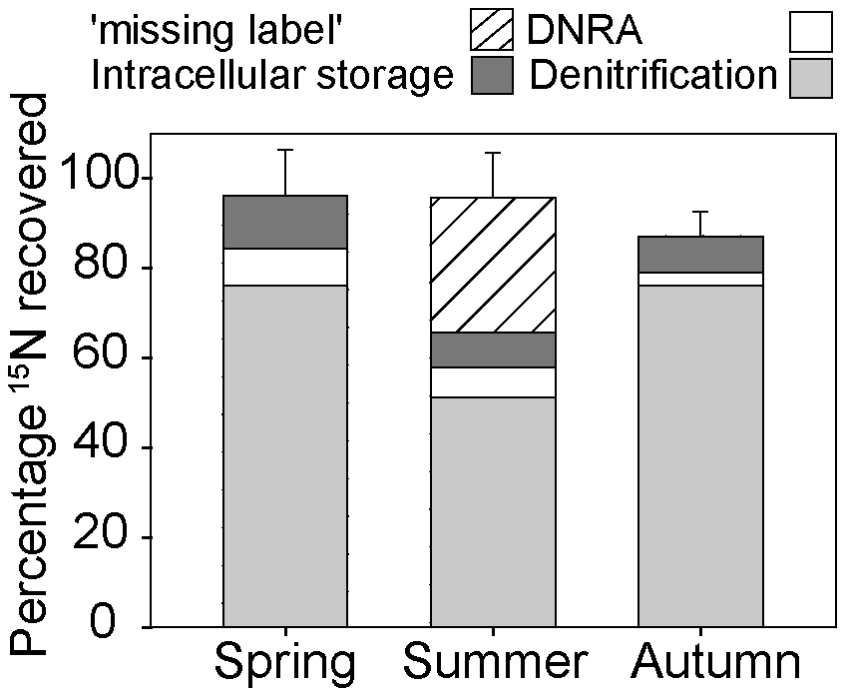
Average percentage of ^15^N recovered in various pools. Denitrification denotes ^15^N that was present as ^29+30^N_2_, DNRA denotes ^15^N that was present as ^15^NH_4_^+^ and intracellular storage represents ^15^N which was present as either ^29+30^N_2_ or ^15^NH_4_ at the termination of the secondary incubation to which no additional ^15^NO_3_ was added. The ‘missing label’ fraction refers to the discrepancy between added ^15^NO_3_^−^, measured ^15^NO_3_^−^ and ^15^N_2_ production (shown in Fig. 4). Error bars are overall SD (n = 3)

Taken together, the *in situ* intracellular nitrate concentrations and the eukaryote-associated nitrate respiration suggested that in all seasons intracellular nitrate storage may have played an important role in the ^15^NO_3_^−^ amendment experiment. This was further supported by a ^15^N mass balance, calculated after all the ^15^NO_3_^−^ within the incubation had been consumed and denitrification and DNRA had ceased. The mass balance revealed that the production of ^15^N-N_2_ and ^15^NH_4_^+^ was insufficient to account for the amount of nitrate consumed; in fact between 20 and 40% of the added ^15^NO_3_^−^ was missing from the porewater and might have entered an intracellular pool (Fig. 6). Nitrate uptake into intracellular pools could have occurred actively, but could also have resulted from isotope equilibration effects between the added ^15^NO_3_^−^ and pre-existing intracellular ^14^NO_3_^−^ pools (as observed in summer; Fig. 4). Similar equilibration effects have recently been observed in sediment incubations carried out with ^15^NO_3_^−^ when stores of intracellular nitrate were present [22], [35].

To investigate whether part of the ^15^NO_3_^−^ added to the sediment had been transferred to an intracellular pool, we ran a second incubation using the sediment cores that were previously amended with ^15^NO_3_^−^. In this subsequent experiment, we percolated the sediment core with oxic seawater that had no added ^15^NO_3_^−^ and then examined whether ^15^N-labelled substrates appeared in the porewater. In the incubation to which no ^15^NO_3_^−^ was added, up to 100% of the “missing” ^15^N appeared in the porewater as 15N-N_2_ or ^15^NH_4_^+^ (Fig. 5a). This indicates that denitrification and DNRA were carried out using a pool of ^15^N that had been stored within an intracellular nitrate pool.

When denitrification and DNRA rates were derived from the incubation with no ^15^NO_3_^−^ added to the porewater, rates of both processes were much lower than in the ^15^NO_3_^−^ amendment experiment, indicating that only a part of the community contributed to the respiration of stored nitrate (Fig. 5c-f). The eukaryote community continued to respire ^15^NO_3_^−^ at the same rate as in the ^15^NO_3_^−^ amendment experiment, whereas prokaryotic DNRA and denitrification rates dropped by 60% and 97% respectively, when compared to the ^15^NO_3_^−^ amendment experiment. This suggests that while eukaryotes were supplied within ample nitrate from the intracellular pool, prokaryote nitrate respiration was limited, possibly by the supply of nitrate leaking out of eukaryotes. As prokaryote denitrification rates dropped so drastically in the incubation where no ^15^NO_3_^−^ was added to the porewater, we were able to observe low rates of eukaryotic denitrification of ^15^NO_3_^−^, which had not been apparent before. It is likely that eukaryotic denitrification also occurred in the ^15^NO_3_^−^ amendment experiment, however it was masked by the comparatively high prokaryote denitrification rates.

Eukaryotes therefore seem to play an important role in intracellular nitrate storage and subsequent nitrate reduction. However, upon inhibition of the eukaryotic community within the ^15^NO_3_^−^ amendment experiment and the incubation to which no ^15^NO_3_^−^ was added, ^15^N storage decreased, but did not cease entirely. Can this fraction of stored nitrate therefore be attributed to prokaryotes? Nitrate storage in prokaryotes is mainly known to occur in large vacuoled sulfur bacteria [32], which are not present in substantial numbers in permeable Wadden Sea sediments. Therefore, the intracellular storage of ^15^N observed after eukaryote inhibition suggests that we had either failed to completely inhibit the eukaryotic community (in which case, prokaryotic rates of DNRA and denitrification derived from intracellular storage would be overestimated), or that nitrate was stored within the sediment by an unidentified prokaryote.

### Implications for N-loss from permeable sediments

Our results illustrate that the nitrate transformations which take place within intertidal permeable sediments are the result of denitrification, DNRA, and intracellular nitrate storage by eukaryotes as well as prokaryotes. The low nitrification rates observed, in combination with the continuous supply of nitrate from the water column make it unlikely that coupled nitrification-denitrification is a significant process in this environment. When nitrate is advectively transported into permeable sediments during tidal inundation, denitrification is the main process that consumes nitrate, although DNRA and intracellular storage by the eukaryotic community are also significant in determining the fate of nitrate (Fig. 6). The magnitude of each of these processes differs seasonally, seemingly in response to organic matter availability, nitrate availability and the composition and abundance of the eukaryotic community.

Intracellular storage of nitrate complicates nitrogen cycling within intertidal sediments, adding a heretofore unconsidered supply of nitrate. So far, areal N-loss estimates are based on denitrification rates determined during the period where the sand flat is inundated with water and nitrate is advectively transported into the sediment [12]. During exposure the advectively dominated system undergoes a shift towards a steady, diffusion limited state in which oxygen penetration depth drops significantly (<1 cm) and any remaining porewater nitrate should be consumed within 40–100 minutes, at which point N-loss is assumed to cease. However, intracellular nitrate storage could act as a reservoir, providing a supply of nitrate to support anaerobic respiration during exposure. In fact, the occurrence of DNRA during exposure has been indicated previously within Wadden Sea intertidal sand flats, where *in situ* NH_4_^+^ concentrations were higher than would be expected from remineralization alone [15].

We calculated the additional N-transformations that may have occurred during exposure, as a result of intracellular nitrate storage, assuming that the amount stored *in situ* is always similar to that which we measured during autumn 2013 (around 28 mmol m^−3^_sediment_). Average N-loss associated with storage was 0.84 mmol m^−3^_sediment_ h^−1^ and N-recycling by DNRA was 0.26 mmol m^−3^_sediment_ h^−1^. Over 6 hours of exposure this would represent the reduction of 6 mmol N m^−3^ sediment or ∼24% of the stored nitrate pool. Integrating these rates over the top 5 cm of intertidal Wadden Sea sediments would represent an additional 0.5 mmol m^−2^ d^−1^ N-loss and 0.22 mmol m^−2^ d^−1^ N-regeneration by DNRA (assuming the sand flat is exposed for 12 hours daily). Reduction of the stored nitrate pool during the diurnal exposure of the sand flat would therefore add an extra 20% to N-loss estimates on top of that calculated previously for these intertidal sediments (2.48 mmol m^−2^ d^−1^ [12]).

The Wadden Sea has previously been identified as a site of N-loss [12], [78], acting as a sink for high riverine N loads and preventing them from reaching the open ocean, a conclusion strongly supported by this study. Our results indicate that intertidal permeable sediments must also be considered as hotspots of eukaryotic nitrate storage and respiration. Furthermore, while DNRA is of less importance than denitrification in this environment, up to 20% of the nitrate reduction that occurs in Wadden Sea sediments is as DNRA, recycling fixed N to NH_4_^+^ which can then fuel primary production. Previously Gao et al. (2012) estimated that 30% of the total annual N input into the Wadden Sea is denitrified within the sediment. The two to five fold higher denitrification rates we have reported here, combined with the additional N-loss during exposure, indicate that this estimate appears to be conservative and that higher N-losses may occur.

## Supporting Information

Figure S1 **Schematic of the sampling scheme.** Sediment cores were filled with freshly collected, homogenized sediment and left to equilibrate overnight, while site seawater was pumped through them on a simulated tidal cycle. Incubations A-E were then carried out sequentially. As detailed in Methods, seawater bubbled with air was either amended or un-amended with ^15^NO_3_^−^ and percolated through the cores. Then concentrations of ^15^N-N_2_, O_2_, ^15^NH_4_^+^ and ^15^NO_x_ were determined either by membrane inlet mass spectrometry (MIMS) or GC-IRMS. The entire porewater volume within the core was then exchanged and the sampling repeated. Finally the cores were percolated with cycloheximide to stop eukaryote activity and the sampling repeated.
(TIF)

Figure S2
**Breakthrough curves of NaBr in sediment.** Briefly, two sediment cores were packed and left to equilibrate overnight as described in the methods. Seawater amended with 4 mM NaBr was then pumped through the core from the bottom inlet at a rate of 30 ml min-1 (as in the experiment). NaBr was sampled regularly from the top of the core and the concentration determined using an ion chromatograph (761 Compact IC, Metrohm), results of which are shown in the figure. The transport of a passive scalar through a flow chamber can be described by the 1-dimensional scalar transport equation (Bear, 1979; Roychoudhury et al., 1998, Rao et al., 2007), which has the analytical solution:

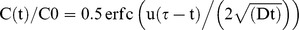
where u is the porewater velocity, τ the residence time, t the time, D the dispersion coefficient and C0 the inlet concentration. The unknown dispersion coefficient is estimated using a least square estimation. The data in the figure represent measurements from two independent cores and show good agreement to the transport model. The estimated dispersion coefficient is 1.04•10^−7^ m^2^/s, which is approximately two orders of magnitude higher than the diffusion coefficient. This is expected since the radial and longitudinal small scale pathways, as well as isolated pockets increase the smearing along the average path in the flow chamber. Compared to the length of the core this effect is less than 50%, which is in the range of previous publications (e.g. Rao et al., 2007). Furthermore, the breakthrough curves show that the outlet concentration of NaBr reaches the value of the inlet water and therefore the porewater in the core is completely exchanged. Upon completion of the breakthrough curve, 600 ml seawater (not amended with NaBr) was pumped through the core at 30 ml min^−1^. Subsequently 12 samples of 6 ml were sampled from the bottom of the core, no NaBr could be detected in these samples.(TIF)

Figure S3 **Oxygen concentrations during the secondary incubation to which no label was added.** The example shown is the same as that in Figure 5a. The dashed line indicates the time at which oxygen was no longer detectable within the sediment, at this point, <90% of the ^15^N could be accounted for. The solid line indicates the point at which 96% of the ^15^NO_3_^−^ could be accounted for in the product pools of ^15^N-N_2_ and ^15^NH_4_^+^.
(TIF)

Table S1 **Volumetric rates of (a) denitrification and (b) DNRA, obtained while oxygen was still present (oxic) and when oxygen had been consumed (anoxic) in the incubations to which ^15^NO_3_^−^ was added.**
(DOC)
