# Supplementary figures and images for: The Fate of Nitrate in Intertidal Permeable Sediments
Source: PLoS One. 2014 Aug 15;9(8):e104517. doi: 10.1371/journal.pone.0104517 (PMC4134218; doi:10.1371/journal.pone.0104517)

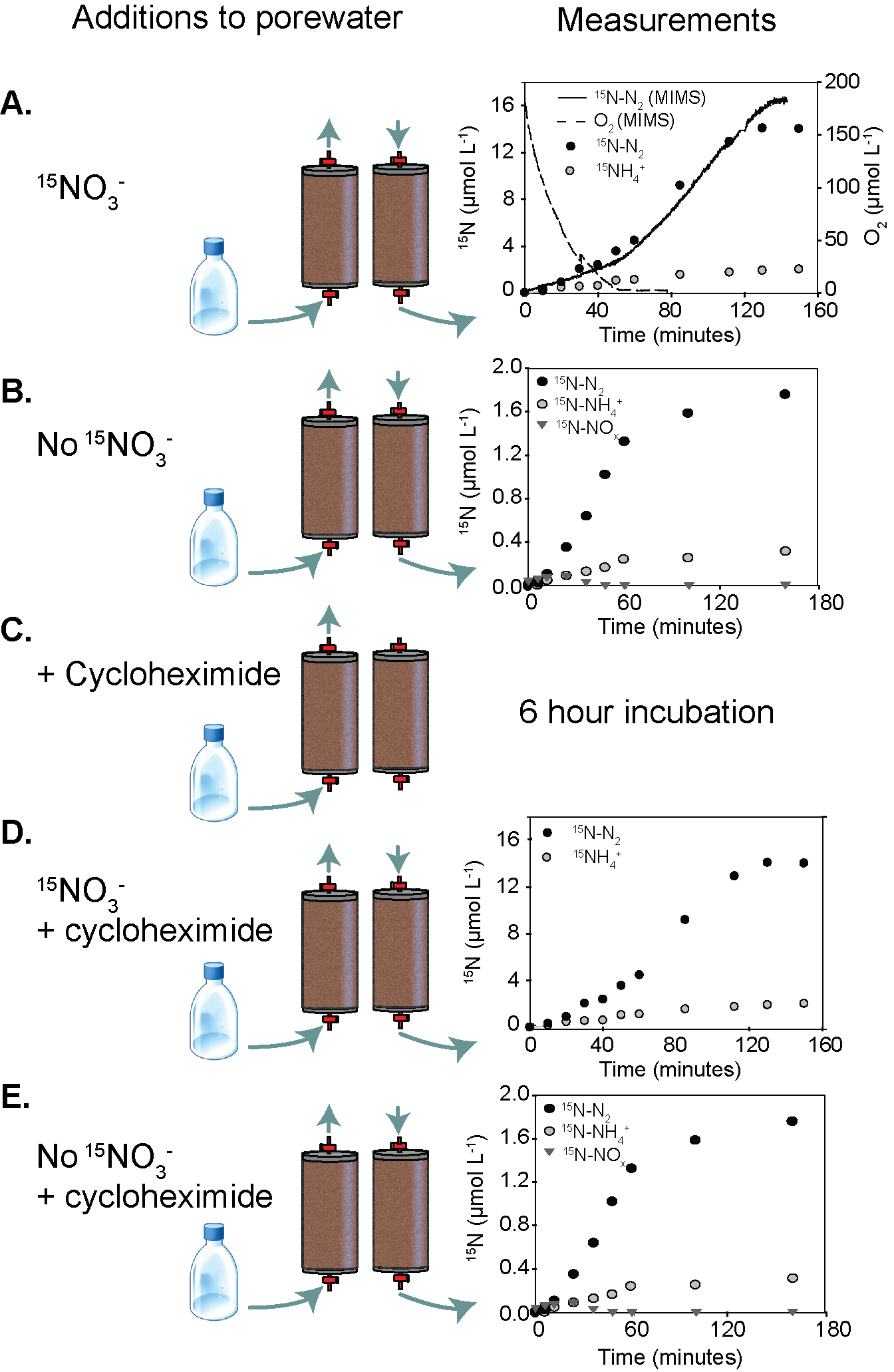

Supplement: Figure S1 — Schematic of the sampling scheme. Sediment cores were filled with freshly collected, homogenized sediment and left to equilibrate overnight, while site seawater was pumped through them on a simulated tidal cycle. Incubations A-E were then carried out sequentially. As detailed in Methods, seawater bubbled with air was either amended or un-amended with 15NO3 − and percolated through the cores. Then concentrations of 15N-N2, O2, 15NH4 + and 15NOx were determined either by membrane inlet mass spectrometry (MIMS) or GC-IRMS. The entire porewater volume within the core was then exchanged and the sampling repeated. Finally the cores were percolated with cycloheximide to stop eukaryote activity and the sampling repeated. (TIF) [file pone.0104517.s001.tif]

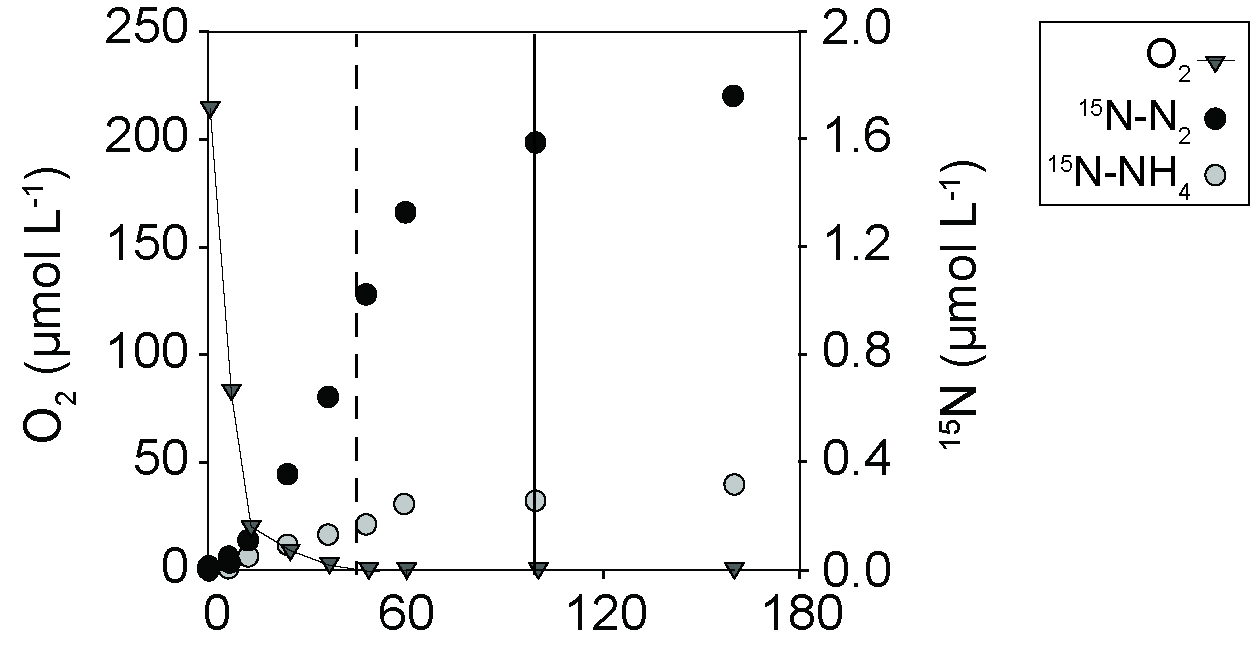

Supplement: Figure S3 — Oxygen concentrations during the secondary incubation to which no label was added. The example shown is the same as that in Figure 5a. The dashed line indicates the time at which oxygen was no longer detectable within the sediment, at this point, <90% of the 15N could be accounted for. The solid line indicates the point at which 96% of the 15NO3 − could be accounted for in the product pools of 15N-N2 and 15NH4 +. (TIF) [file pone.0104517.s003.tif]
