# Supplementary material for: The Fate of Nitrate in Intertidal Permeable Sediments
Source: PLoS One. 2014 Aug 15;9(8):e104517. doi: 10.1371/journal.pone.0104517 (PMC4134218; doi:10.1371/journal.pone.0104517)
Supplement: Table S1 — Volumetric rates of (a) denitrification and (b) DNRA, obtained while oxygen was still present (oxic) and when oxygen had been consumed (anoxic) in the incubations to which 15NO3− was added. (DOC) [file pone.0104517.s004.doc]

|  |  |  | **Replicate** | **Rate (µmol N L^-1^ h^-1^)** | **S.E.** |
| --- | --- | --- | --- | --- | --- |
| **a. Denitrification** | Spring | Oxic | 1 | 5.00 | 0.27 |
|  |  |  | 2 | 7.75 | 0.33 |
|  |  |  | 3 | 7.22 | 0.79 |
|  |  | Anoxic | 1 | 23.52 | 0.44 |
|  |  |  | 2 | 25.09 | 0.21 |
|  |  |  | 3 | 28.71 | 1.11 |
|  | Summer | Oxic | 1 | 2.41 | 0.22 |
|  |  |  | 2 | 1.39 | 0.16 |
|  |  |  | 3 | 1.06 | 0.06 |
|  |  | Anoxic | 1 | 22.89 | 1.12 |
|  |  |  | 2 | 25.81 | 1.16 |
|  |  |  | 3 | 25.46 | 1.66 |
|  | Autumn | Oxic | 1 | 6.41 | 0.09 |
|  |  |  | 2 | 7.71 | 0.28 |
|  |  |  | 3 | 9.34 | 0.23 |
|  |  | Anoxic | 1 | 57.37 | 0.68 |
|  |  |  | 2 | 59.12 | 1.18 |
|  |  |  | 3 | 72.31 | 1.18 |
| **b. DNRA** | Spring | Oxic | 1 | 1.64 | 0.14 |
|  |  |  | 2 | 1.00 | 0.11 |
|  |  |  | 3 | 1.66 | 0.22 |
|  |  | Anoxic | 1 | 4.10 | 0.11 |
|  |  |  | 2 | 3.73 | 0.11 |
|  |  |  | 3 | 4.64 | 0.06 |
|  | Summer | Oxic | 1 | 1.23 | 0.05 |
|  |  |  | 2 | 0.42 | 0.02 |
|  |  |  | 3 | n.d | 0.00 |
|  |  | Anoxic | 1 | 4.04 | 0.02 |
|  |  |  | 2 | 4.27 | 0.11 |
|  |  |  | 3 | 3.29 | 0.27 |
|  | Autumn | Oxic | 1 | 0.73 | 0.06 |
|  |  |  | 2 | 0.31 | 0.01 |
|  |  |  | 3 | 0.91 | 0.02 |
|  |  | Anoxic | 1 | 2.08 | 0.05 |
|  |  |  | 2 | 2.49 | 0.04 |
|  |  |  | 3 | 2.34 | 0.18 |
